# Patients' perceptions of self-management of high blood pressure in three low- and middle-income countries: findings from the BPMONITOR study

**DOI:** 10.1017/gheg.2020.5

**Published:** 2020-07-20

**Authors:** Tala Al-Rousan, M. Amalia Pesantes, Sufia Dadabhai, Namratha R. Kandula, Mark D. Huffman, J. Jaime Miranda, Rafael Vidal-Perez, Anastase Dzudie, Cheryl A. M. Anderson

**Affiliations:** 1 Department of Medicine, University of California San Diego School of Medicine Division of Global Public Health, La Jolla, USA; 2 Department of Medicine, Universidad Peruana Cayetano Heredia, CRONICAS Centre of Excellence in Chronic Diseases, Peru; 3 Department of Epidemiology, Johns Hopkins Bloomberg School of Public Health, Baltimore, USA; 4 Department of Medicine, Northwestern University Feinberg School of Medicine, Chicago, USA; 5 Department of Preventive Medicine, Northwestern University Feinberg School of Medicine, Chicago, USA; 6 Department of Cardiology, Hospital Universitario Lucus Augusti, Lugo, Spain; 7 Clinical Research Education, Networking and Consultancy (CRENC), Yaounde, Cameroon; 8 Department of Family Medicine and Public Health, University of California San Diego School of Medicine, La Jolla, USA

**Keywords:** Cameroon, hypertension, Malawi, medication, perception, Peru, qualitative, self-management

## Abstract

Hypertension is the leading risk factor for global disease burden. Self-management of high blood pressure (BP) through self-monitoring and self-titration of medications, has proved to be one successful and cost-effective tool to achieve better BP control in many high-income countries but not much is known about its potential in low- and middle-income countries (LMICs). We used semi-structured questionnaires and focus groups in three LMICs; Peru, Cameroon and Malawi to examine perceptions and attitudes of patients diagnosed with essential hypertension towards living with hypertension, BP measurement and treatment, patient–physician relationship and opinions about self-management of high blood pressure. Results in all three countries were comparable. Patients showed varied levels of health literacy related to hypertension. BP measurement habits were mostly affected by resources available and caregiver support. Treatment and adherence to it were primarily affected by cost. Most patients were welcoming of the idea of self-management but skeptical about the ability to do self-monitoring accurately and the safety involving self-titration of medications.

## Introduction

High blood pressure remains the leading preventable cause of premature death and disability worldwide, killing almost eight million people every year and is projected to increase by 60% to affect 1.6 billion adults worldwide by 2025[1,2]. While high-income countries have stable or decreasing rates[3], hypertension prevalence rates are increasing in low- and middle-income countries (LMICs)[4] and control of BP in these countries continues to be poor, often less than 10%[5]. Prevalence rates vary widely in these countries, from 11% to 37%[6]. These trends create a need for more foundational research to understand the global disparities of hypertension to inform health policies and interventions[7–9].

Self-management of high blood pressure interventions include an array of activities including self-monitoring and lifestyle changes. Home blood pressure monitoring (HBPM) and self-titration of medications have proven to be cost-effective self-management strategies to control BP in higher-income countries[10–12]. While there is growing evidence on the premise of community-wide interventions in reducing the incidence of hypertension in LMICs[13], HBMP and self-titration of medications as a BP self-management strategy have been associated with health behavior and lifestyle modifications to achieve optimal BP control[14–17].

In this study, we aimed to understand current BP management practices in LMIC settings, as well as the feasibility of utilizing HBPM and self-titration of medications in three LMICs. We conducted a qualitative study examining hypertensive patients' experiences with hypertension and perspectives on self-management of BP. We reported and compared patients' attitudes and perspectives towards the diagnosis, measurement, and treatment of hypertension, and thoughts on HBPM and self-titration of medications.

## Methods

The BPMONITOR study was a multi-country pilot study developed to understand the feasibility of HBPM and development and execution of a stepped care self-titration medication plan with the treating doctor according to standard of care. The goals and protocol of BPMONITOR have been reported elsewhere[18]. In this qualitative study, we used individual in-depth interviews in Peru and focus group discussions (FGD) in Cameroon and Malawi with patients diagnosed with hypertension, as well as their caregivers and family members. We adhered to the Criteria for Reporting Qualitative research (COREQ) guidelines in our reporting.

### Setting

Data were collected between June 2016 and May 2017 in Lima, Peru, from March 2016 to May 2016 in Douala, Cameroon, and from October 2016 to February 2018 in Blantyre, Malawi using a standardized protocol and maximum variation sampling. In Peru, participants were recruited from cardiology and geriatric outpatient departments affiliated with Hospital Santa Rosa, a public tertiary hospital managed by the Ministry of Health located in the city center that serves a population of 67 000 people from five districts of Peru. In Cameroon, participants were recruited from the cardiology outpatient department from Deido District hospital, a public and primary care facility which serves the entire Deido district and four neighboring (i.e. Akwa, Bonamoussadi, Makepe and Bonaberi). In Malawi, participants were recruited from the hypertension outpatient department at Queen Elizabeth Central Hospital, a public hospital providing free services to more than 100 000 from surrounding areas. All three facilities are tertiary hospitals serving patients to better capture the diversity and heterogeneity between countries and not within them. These hospitals primarily serve low-income people in urban areas and tend to have high patient volumes, for which HBPM and self-management, including self-titration, might help improve BP control. Patients participated in this study while they were waiting for their appointments or after they have completed them.

### Participants

Adults diagnosed with essential or primary hypertension who had access to healthcare were recruited to participate in the study. To be eligible for study participation, a person had to be: (1) 18 years of age or older; (2) diagnosed with hypertension or a caregiver of a patient with hypertension; and, (3) able to voluntarily and independently consent to participate in the study. At each study site, an equal representation of men and women were sought, as well as time since diagnosis using a 1, 5 or 10 year threshold, given that there is evidence that health-seeking behaviors[19], self-management practices vary according to chronic conditions[20, 21] and the social support structure differ by gender. In Peru, 16 of 32 patients who have been invited to participate in the study completed the in-depth interviews. In Cameroon, 16 participants were invited to and completed the study. In Malawi, 44 participants were approached by the study team and 10 declined to participate. In total, 16 interviews in Peru, four FGDs in Cameroon and one FGD in Malawi were conducted.

### Data collection

**Fig. 1.**
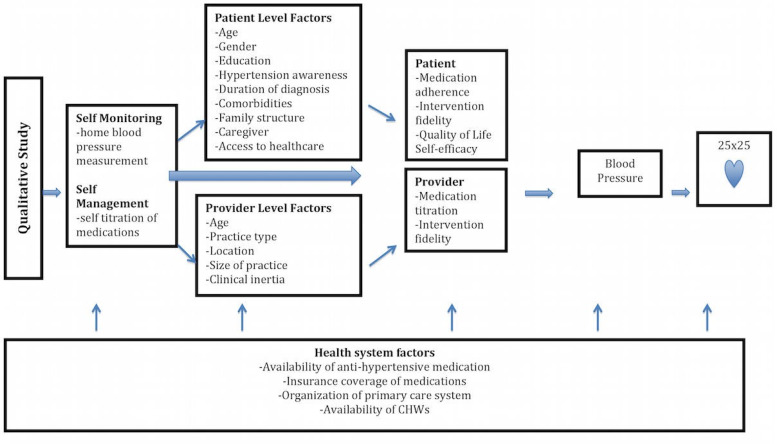
Conceptual framework describing the relationship between home blood pressure management context and blood pressure

The research team designed and piloted a semi-structured focus groups guide (Table 1) for conducting the interviews and FGDs with participants and caregivers. This guide was developed using a conceptual model (Fig. 1) and covered experiences and attitudes towards having high blood pressure, measurement (regularity and approach), treatment (adherence and access), patient–physician relationship, and opinions about monitoring their blood pressure at home and potentially self-titrating their medications based on previous research in high-income countries[18, 22].

**Table 1.** Semi-structured interview guide for patients and their family caregivers

*A. Overall experience and attitudes*
A1. Does high blood pressure worry you? Tell me why
*B. Measurement*
B1. Do you get your blood pressure regularly checked?
*Probe*: If yes to B1, ASK: Where do you get it checked? Why do you get it checked? Are there any difficulties in getting your blood pressure checked?
*Probe:* If yes to B1: Does anyone in your family help you check your blood pressure?
*Probe*: If they say no to B1, ASK: Why? What stops from you getting it checked?
*C. Treatment*
C1. Are you taking medication for your blood pressure?
*Probe:* Are there things that make it difficult for you to take your medicine?
*Optional Probe*: How do you pay for your blood pressure medication?
*D. Patient–physician relationship*
D1. When your doctor gives you advice about high blood pressure or prescribes medicine for your high blood pressure, do you ever consult other people, family, friends, or other doctors, before following the doctor's advice?
*E. General description of BP MONITOR Intervention (develop medication plan with the doctor, get home monitor, self-monitor, record readings, and titrate medicine)*
E1. Would you be interested in participating in such a program/intervention?
*Probe* - Why? How do you think this could benefit you? Is there anything that worries you?
*Final Thoughts*
F1. What would make life better for people with high blood pressure?

Participants first completed a short questionnaire designed to collect socio-demographic data including age, gender, marital status and years of education. Mean interview time mean was 45 min (s.d, 17 min) and FGDs was 30 min (s.d., 25 min). In Peru, Interviews were conducted by a female research assistant with a degree in communications and previous experience in qualitative research. In Cameroon, FGDs were conducted by a male research assistant with previous training in qualitative data collection. In Malawi, focus group discussions were conducted in the native Chichewa language by two male research assistants (one primary and one as a backup) with clinical training and previous training in qualitative data collection; patients in the hypertension were approached as a group with a ‘waiting room talk,’ with individual follow-up with anyone expressing interest to join. All interviews were conducted in the local language at the site, performed at the health facilities and recorded, after obtaining written consent from all participants. In all three sites, data collection continued until saturation was reached where no new themes emerged[23].

### Data analysis

Data from focus groups and in-depth interviews were put into matrices organized around the predefined six topics in the interview/focus group guide (Table 1). We used both inductive and deductive approach to thematic analyses where multiple experiences and perspectives contribute to developing the final research outcome[24–26]. Interview recordings were listened to and charted in the original language into matrices according to the predefined topics, which were translated and shared with two other researchers to ensure accuracy in the interpretation of the data. Relevant quotes for each topic were selected and then two separate researchers per site organized the information for analysis and interpretation. The second author (MAP) created the initial code book for each group. Using the initial code book, three coauthors (MAP, TA, NK) coded focus group transcripts from Peru, Cameroon and Malawi using Microsoft Word. The coders (MAP, TA) adopted line by line coding to capture emerging themes using a mix of descriptive and conceptual codes and refined the codebooks through weekly discussions. Codes were reflected in the themes that are included in the results section.

## Results

### Characteristics of participants

Characteristics of study participants are shown in Table 2. In Peru and Malawi, reasons for not participating in the study were: (1) patients were concerned about missing their appointment if the interview was held while they were waiting to see their cardiologist, or (2) because they needed to go back home to attend personal matters and could not stay longer at the hospital after their appointment has ended. The number of caregivers included was one in Peru, three in Malawi (one mother, one father and one grandmother) and none in Cameroon. In Cameroon, 30 participants were invited to participate and four declined due to time constraints. One FGD in Malawi included females only, while the other three included males and females; the goal of a single all-male focus group discussion was not achieved due to men being concerned about time away from work.

**Table 2.** Demographics of participants included in the study

Demographic	Peru (*n* = 16)	Cameroon (*n* = 26)	Malawi (*n* = 34)
Age, years (median, IQR)	64.0 (60.8–68.3)	62.0 (54.3–69.5)	50.9 (38.3, 61.0)
Gender (female, %)	50.0	50.0	58.8
Marital status (married, %)	25.0	60.0	70.0
Education (high school, %)	68.8	50.0	38.0
Time since diagnosis, years (Median, IQR)	8.0 (3.0–20.0)	4.0 (1.0–7.8)	4.2

IQR, interquartile range.

### Experience and attitudes towards living with high blood pressure

In all three countries, when asked if high blood pressure worries them, participants varied in the degree to which they were concerned about having high blood pressure. Some explained that they feel ‘fear’ and ‘anxiety’ associated with their hypertension. Concerns ranged from specific conditions in the present, such as dizziness and symptoms exacerbated by hot weather such as leg swelling, to broad concerns about the risk of ‘sudden, catastrophic’ health events in the future, such as a stroke, that may lead to loss of work. There were several references to confusion experienced when their readings vary within the same day, which may lead to some distrust in the medication regimen that they were following. In Peru, some participants felt that blood pressure only rises occasionally after they eat a ‘meal full of carbohydrates’ or when they feel ‘stressed out’. Several individuals voiced that they were ‘not anxious’ since hypertension is manageable and treatable with proper diet, exercise and medication as prescribed by health care workers. A restricted diet may be stressful to some, particularly with few options for food diversity voiced by the Malawi's participants. There was a strong acknowledgement of the role of the health care worker in management of hypertension. Some variation in responses to the question ‘Does high blood pressure worry you?’ are shown in Table 3.

**Table 3.** Responses and representative quotes to the question: ‘Does high blood pressure worry you?’

Country	Relevant quote
Peru	*‘I could have a heart attack, a stroke. I could very well die and become hemiplegic’. (Male, 63 Years old)*
Cameroon	*‘Hypertension doesn't worry me, in fact why should it? It was only my hand that had a problem and when I came to see the doctor she told me I have hypertension.’ (FGD3)*
Malawi	‘*Complications of high BP like stroke are life changing events resulting at times in loss of work and changes in speech.’(FGD2)*

### Measurement of blood pressure

When asked about monitoring their blood pressure, participants spoke about the places where they get their blood pressure measured, the frequency and their reasons for doing it, reasons for measuring their BP and difficulties getting it checked. Only a few participants at each site mentioned that they were monitoring their BP at home (33.3% of patients in Peru, 11% in Cameroon, 6.4% in Malawi) with the majority only relying on getting their BP measured only during pre-scheduled clinic appointments (weekly, monthly or every 3–6 months appointments). There were concerns raised about the lack of resources to monitor their blood pressure either at home or at the clinics. Among those who monitored their BP at home, the frequency of home BP monitoring seemed to be low and tied to either: (1) their belief that they are adherent to their medications, thus there was no need to check their BP, (2) the fact that their doctor did not instruct them to do so, or (3) they feel unwell on a certain day which makes them check their BP. Of these, 4 out of 15 participants in Peru said that they measure their BP in other places such as pharmacies located near their home or healthcare facility which were private and managed by the district municipality. They mostly do so because they want to either confirm their BP readings which were measured at the clinic, or check their BP more frequently. Complaints about the costs associated with getting their BP checked outside the clinic or several times were brought up in the discussions. When asked ‘Does anyone in your family help you check your blood pressure?’ only those who were likely to monitor their BP at home expressed a positive role for peers or family members in encouraging more frequent home BP measurements. Examples of relevant quotes from participants are shown in Table 4.

**Table 4.** Responses and representative quotes to the question: ‘Are there any difficulties in getting your blood pressure checked?’

Country	Relevant quote
Peru	*‘I measure my BP at the pharmacy where I buy my medication, there I know the owner, who doesn´t charge me for this service.’(Male, 70 Years old)*
Cameroon	*‘She does not have someone at home to take her BP neither is there a health center nearby where she can measure her BP’. (Caregiver. FGD2)*
Malawi	*‘‘We are sent back from health centers because of lack of equipment. Also, if we go to a private clinic, we should pay a lot of money.*’ (FGD3)

### Treatment of high blood pressure

When asked ‘are you taking your medications for blood pressure?’ participants revealed that they mostly do. In Peru, two of the 16 patients expressed that they do take more than one pill of one medicine when they are feeling specifically ill that day. In Malawi, patients described that the high pill burden ‘for those on treatment for other chronic diseases, such as HIV,’ are lead causes for their missed dosages. Participants expressed a linkage between access and adherence to medications. Reasons for compromised adherence fell into four categories; (1) unintentional missed doses, (2) access issues to medications and healthcare, (3) health beliefs and (4) medication side effects. In Peru, two out of the 16 participants stated that another perceived limitation is when the doctor is on vacation or occasionally when they are on strike. After a doctor's holiday or strike, it is harder to obtain an appointment because the quotas per doctor are filled faster. Patients described two sources of access issues. Medication stock-outs were a significant concern although there is an agreement that, ‘*medications should be available’* (Malawi). One of the 31 participants in Malawi described that he is on one antihypertensive agent only because the others that were prescribed to him are out of stock. Additionally, there were challenges with resupply when traveling and not having their medical records on them (either lost or not properly documented), and when they are unsure of drug names. Example quotes from three countries are shown in Table 5. When asked how they pay for their medications, participants in all three countries explained frustration at how expensive many drugs are especially when they are not covered by the national health insurance. Participants secured these costly medications through mostly three ways: (1) direct financial support from their family, (2) loans, or (3) minor income-generating activities to cover the direct cost of medications. Representative quotes from the three countries are shown in Table 6.

**Table 5.** Responses and representative quotes to the question: ‘Are you taking medications for your blood pressure?’

Country	Relevant quote
Peru	*‘Besides taking my pills at the time indicated by the doctor, I take one pill when I have a headache and feel that my blood pressure has gone up. There have been times where I have taken 4 pills in one day.’ (Female, 56 Years old)*
Cameroon	*‘When I am really ‘taken’ by the condition. I take medications but I have not taken any medication for the last month because I feel okay.’ (FGD3)*
Malawi	*‘Even when you are started on medications, the blood pressure is dynamic going up and down; because of that the value of taking medications is not entirely clear.’ (FGD1)*

**Table 6.** Responses and representative quotes to the question: ‘How do you pay for your blood pressure medications?’

Country	Relevant quote
Peru	*‘They don´t give me Valsartan [at the public pharmacy] and I have to buy it. When I don´t have enough money, I buy Nifedipino of 30 that is the generic one.’ (Female, 62 Years old)*
Cameroon	*‘I try to buy my medications; sometimes I buy three packets that can last me 3 months, but if I don't have the means, I buy a packet of 30 tablets that will last me at least a month.’ (FGD4)*
Malawi	*‘At times, we are only able to afford medications for half of the month due to cost, when told to source them privately.’ (FGD4)*

### Patient–physician relationship

Many participants felt that their provider interactions are not thorough enough in terms of either physical assessments or level of discussion and counseling while a few explained that they had developed a close relationship with their doctor over the years and described that as ‘friendship.’ Almost quarter of the participants in Malawi specifically highlighted the lack of continuity in service provision by the same clinician as a challenge in understanding the status of their hypertension. In Peru, almost 50% of the study participants relied on the information provided by their doctor for managing their BP. They stated that they did not seek health advice from other people or looked for information from other sources while in Cameroon and Malawi, the Internet and consulting with peers was a go-to source for some participants. In Cameroon, some patients explained that they had developed a close relationship with their doctor over the years, which in some cases is described as ‘friendship.’ Participants stated that when they have doubts about anything such as having new symptoms, they ‘simply’ ask their doctors. In Peru, four patients said that they have never been told exactly what is hypertension or its associated risks if not managed well. Some patients reported that he had never been asked to monitor his BP on a daily basis.

### Opinions about self-management and medication self-titration

A broad range of key themes ranging from patient empowerment and engagement to seeing no added value emerged when an intervention that includes HBPM and self-management, including self-titration of medications was proposed. The majority of participants agreed that the intervention would allow people to take more responsibility for their disease progression, specifically ‘motivating’ patients to engage in care more consistently, and increase control of their BP. Some felt that HBPM could serve as an early warning system to visit a health facility sooner than later in case of abnormal readings, which could lead to quicker attention to disease progression and medical care. However, there was a minority in both Malawi and Cameroon that saw no value in home BP monitoring since the readings will likely be ‘*the same like those you get at the doctor's office.’* Participants voiced that a shift to home BP monitoring could reduce burden on both patients and the healthcare system. On a patient-level, patients thought that home BP monitoring reduces the need to go to clinic replacing that with calls to the doctor's office which thus saves them transportation money, time and effort. On a system-level, participants stated a possible reduction in the number of patients going to and waiting in clinics just to get their BP checked, which improves the ‘efficiency’ of the entire healthcare system.

Concerns about an intervention that includes HBPM revolved around three main issues. First, for a few, the intervention sounded burdensome, of no or a small value or time-consuming. This may be indirectly linked to another theme that emerged around a perceived increase in stress and anxiety in the face of self-management BP. Second, two practical challenges provided by the participants included the lack of adequate numbers of clinicians to receive calls from every patient; and a concern that the intervention would only last for a period of time and then the current state of BP care and control would resume. Third, some expressed that it would be a form of injustice to the other patients who are not to be included in the study since they will not be able to self-monitor their BP. When asked ‘what worries you about the intervention,’ participants expressed associated costs, risks of self-titration by doing ‘mistakes’ as the main issues that worry them (Table 7).

**Table 7.** Responses and representative quotes to the question: ‘Is there anything that worries you about self-management?’

Country	Relevant quote
Peru	*‘Give everybody the device so that they can measure their blood pressure themselves, like in the United States.’ (Male, 55 Years old)*
Cameroon	*‘I think that the part of patients adjusting their medication will be risky because they may take more than is required.’ (FGD1)*
Malawi	*‘What if I get the readings mixed up, or the new medication orders.’ (FGD2)*

## Discussion

In three LMIC settings, Peru, Malawi and Cameroon, we found unique distinctions in the context in which HBPM and self-management, including self-titration may be used to improve BP control. These distinctions were clear in the experiences and attitudes related to hypertension, measurement of BP, patient–physician relationship and feasibility of future HBPM interventions.

With regard to experiences and attitudes related to the diagnosis of hypertension, we found that in all three countries, there is a clear role for health literacy and the source of information that the patients primarily rely on in shaping their perceptions of illness, treatment and future HBPM interventions. A potential HBPM strategy in this case may include focusing on increasing awareness by engaging providers and caregivers as well and paying attention to certain dietary and cultural attributes to BP control that differ at each setting. Together lifestyle modifications and self-management through HBPM and self-titration of medications achieve better BP control[17].

Measurement of BP was closely concomitant with what is available and affordable. Some participants expressed that they have not adhered to measuring their BP regularly because their physicians have not asked them to. These patients were not likely to self-monitor their BP or see the value in doing so. This is in contrast to a meta-analysis paper that identified disagreement with clinical recommendations as the most common barrier among health care providers[27]. It is impossible to tell whether a similar sort of disagreement would be a barrier to HBPM in these patients or whether adherence would improve if providers' feedback and recommendations were part of HBPM interventions.

The role patient–physician relationship plays in hypertension care and medication adherence is well established in the literature[28, 29]. Patients' satisfaction resulting from building the relationship and empathy with physicians appears to be associated with medication adherence among hypertensive patients[30]. Moreover, a patient-centered, multidisciplinary team has proved to be a key feature of effective care models that have been found to improve care processes and control rates globally[31]. Our findings, by large, indicate a clear trust in advice received by patients from their doctors. However, the health system in these countries seem to be suffering lack of resources which may negatively affect the patient–physician relationship. This is obvious in the case of Peru where doctors can be out on strike for a period of time, or the long waiting time in limited-resource settings where the ratio of doctor to patients is low[32]. A successful implementation of HBPM strategies in these resource-limited settings must bear in mind the dynamics of patient–physician relationships in local contexts.

Although self-management is a relatively ‘new’ concept for many LMICs health systems, this study has found that patients are mostly welcoming of the idea had they been educated on the details of the intervention and on hypertension[14]. Engaging patients in the management of their disease is a growing trend in chronic disease management like in the case of diabetes; another very common chronic condition in LIMCs[33, 34]. A recent meta-review of both quantitative and qualitative systematic reviews of self-management for people with hypertension highlighted that strategies for self-management are complex and are generally welcome by patients[35]. Self-titration of medications seemed to worry some patients since some lacked belief in their own ability to follow instructions and not make mistakes. In previous studies, the reluctance to change medication for borderline readings suggested behavior similar to the clinical inertia seen for physicians in analogous circumstances. Additional support for patients and physicians to implement prearranged medication changes may allow for more effective self-management[22]. A systematic review using data from almost a million and a half adults from 45 LMICs estimated the overall prevalence of hypertension to be 32.3% with the highest rates being among the elderly, overweight, non-educated and urban settlers[6]. Recent data from Mexico and Honduras found, on average, a 4.2 mm Hg reduction in BP in groups that use a home BP monitor combined with automated interactive voice response messages, in comparison to controls[36]. There is evidence from Malaysia, another LMIC, that perceptions, attitudes and knowledge play a major role in self-management of hypertension[37].

Doing a multi-site study across three very different countries using three different languages to collect data introduces certain limitations to this study. Some nuances might have been lost in the translation. However, the opportunity to discuss our findings have reduced the possibilities of misinterpretation of the data. First, the demographic data was self-reported. Since the study was designed to establish a baseline understanding about perceptions, a limited number of questions were included in the study protocol. The choice of conducting semi-structured interviews or focus group discussions at each study site was dictated by the time allowed without affecting the clinic's flow at each site and bearing in mind the time conflicts for participants. The number of focus groups was small, yet Guest *et al*., suggest that more than 80% of themes in qualitative studies are discoverable within two to three focus groups[38]. Also, another limitation is the time allocated by interviews which may have been short due to feasibility issues. Both data collection methods are believed to generate a similar number of unique items and can be used interchangeably to study perceptions[39]. FGs risk having the more vocal participants override the other voices. Moderators addressed this issue by encouraging inactive participants to contribute to the discussion. Additionally, these results may not be generalizable to all patients living in these heterogeneous geographic locations such as those living in more rural parts or being seen in primary or community care settings. Lastly, self-management practices vary according to being diagnosed with other comorbidities and we plan to collect this data and incorporate into future work.

## Conclusion

This study presents foundational research that explores patient attitudes and perceptions of hypertension diagnosis, measurement, treatment, patient–physician relationship and self-management of blood pressure in three different LMICs. We also examined the readability of introducing HBPM in these different parts of the world drawing on patients favoring HBPM and a medication self-titration plan to address cost as long as safety is taken into consideration. Our study revealed important knowledge gaps in the causes and consequences of hypertension, BP measurement and adherence to treatment that need to be addressed before introducing a HBPM intervention. There is a critical need to understand the local context at each setting and identify the best ways to promote HBPM practices to ultimately achieve better BP control.
